# Anti-VEGF Treatment Strategies for Wet AMD

**DOI:** 10.1155/2012/786870

**Published:** 2012-02-28

**Authors:** Jaclyn L. Kovach, Stephen G. Schwartz, Harry W. Flynn, Ingrid U. Scott

**Affiliations:** ^1^Department of Ophthalmology, Bascom Palmer Eye Institute, School of Medicine, University of Miami, 311 9th St North, Naples, FL 34102, USA; ^2^Penn State Hershey Eye Center, Hershey, PA 17033, USA

## Abstract

Over the past few years, antivascular endothelial growth factor (VEGF) therapy has become a standard treatment for neovascular age-related macular degeneration (AMD). During this time, treatment strategies have evolved from a monthly dosing schedule to individualized regimens. This paper will review the currently available anti-VEGF agents and evidence-based treatment strategies.

## 1. Introduction

Age-related macular degeneration (AMD) is the result of complex interactions between lipofuscinogenesis, drusenogenesis, and inflammation which can lead to choroidal neovascularization (CNV). Unregulated choroidal angiogenesis begins when local inflammation, and potentially ischemia, disrupt a delicate interplay between numerous stimulators and inhibitors, which may lead to an imbalance between the proangiogenic vascular endothelial growth factor (VEGF) and the antiangiogenic pigment epithelium-derived factor (PEDF) [1]. VEGF-A plays an important role in angiogenesis and vascular permeability. The *VEGF-A* gene is organized into eight exons on chromosome 6p21. Alternate gene splicing can generate 9 isoforms, the most prevalent of which is VEGF_165_. VEGF-A (or VEGF as it is commonly known) is a dimeric glycoprotein that interacts with two tyrosine kinase receptors, VEGFR-1 and VEGFR-2 located primarily on endothelial cells [2]. 

Animal studies have demonstrated that VEGF overexpression in the retinal pigment epithelium (RPE) leads to CNV [3]. In a monkey model of laser-induced CNV, intravitreal injections of an anti-VEGF-A antibody prevented the development of CNV and reduced leakage from preexisting CNV [4], and intravitreal injections of ranibizumab (Lucentis; Genentech/Roche, South San Francisco) in combination with photodynamic therapy (PDT) with verteporfin (Visudyne; Novartis, Basel, Switzerland) decreased CNV leakage and induced CNV regression [5].

Over the past few years, anti-VEGF therapy for neovascular AMD has become a standard treatment for neovascular AMD. This paper will review anti-VEGF treatment options and current treatment strategies.

## 2. Pegaptanib

Pegaptanib (Macugen; Eyetech, Palm Beach Gardens, FL) is a selective inhibitor of VEGF_165_. The Vascular Endothelial Growth Factor (VEGF) Inhibition Study in Ocular Neovascularization (VISION) Study included two concurrent, prospective, randomized, double-masked, dose-ranging, controlled phase III clinical trials that demonstrated that intravitreal administration of pegaptanib at 6-week intervals for 48 weeks reduced the chance of moderate and severe vision loss in patients with neovascular AMD regardless of angiographic subtype of CNV. In the group that was given the 0.3 mg dose of pegaptanib, 70 percent of patients lost fewer than 15 letters of visual acuity, compared with 55 percent among the control group. More patients receiving 0.3 mg pegaptanib compared to sham maintained or gained visual acuity (33 percent versus 23 percent) [6]. Patients who continued on pegaptanib during the second year of the VISION Study lost less visual acuity compared to those who discontinued pegaptanib or remained on PDT or no treatment [7].

## 3. Bevacizumab

**Figure 1:**
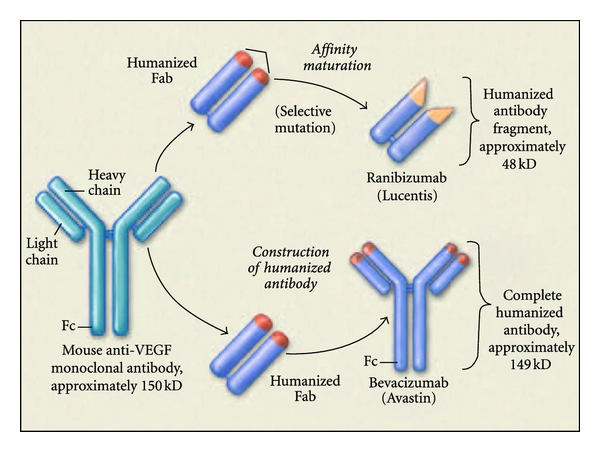
Ranibizumab is a recombinant humanized monoclonal antibody fragment. Bevacizumab is a recombinant humanized IgG antibody. Both bind to and inhibit all biologically active forms of VEGF-A and are derived from the same mouse monoclonal antibody. Ranibizumab has been genetically engineered to bind with higher affinity than bevacizumab (see [8]).

Bevacizumab (Avastin; Genentech/Roche, South San Francisco) is a full-length, humanized, monoclonal antibody with two VEGF-A binding sites (Figure 1). In 2004, the antiangiogenic effects of bevacizumab led to its FDA approval for the treatment of metastatic colon cancer [10]. A potential role for bevacizumab in the treatment of AMD was established when animal studies revealed that fluorescein-conjugated bevacizumab leaked from laser-induced CNV after systemic administration to cynomolgus monkeys, suggesting that systemic bevacizumab could leak from CNV in patients with AMD and competitively inhibit extravascular VEGF [11]. The Systemic Avastin for Neovascular AMD (SANA) Study was an open label prospective clinical study that evaluated the safety, efficacy, and durability of bevacizumab for the treatment of subfoveal CNV in AMD. Participants were treated at baseline with an infusion of bevacizumab (5 mg/kg) followed by one or two additional doses at two-week intervals. At 24 weeks, systemic bevacizumab was well tolerated and associated with an average of a 14 letter gain in the best-corrected visual acuity (BCVA) and a reduction in central retinal thickness by 112 microns on optical coherence tomography (OCT) in the 18 patients studied [12]. Large clinical trials of intravenous bevacizumab were not pursued due to the perception that intravitreal therapy would be a safer alternative.

The first reported case of bevacizumab injected into a human eye was described in a 2005 case report of a 63-year-old woman with subfoveal, predominantly classic CNV. Four weeks after a single-intravitreal injection of bevacizumab (1 mg), resolution of subretinal fluid on OCT was noted, and no adverse effects were observed [13]. The safety and efficacy of intravitreal bevacizumab were investigated in a retrospective case series of 79 patients treated with monthly bevacizumab (1.25 mg) until resolution of macular edema, subretinal fluid, and/or pigment epithelial detachment (PED) as observed on OCT. After two months, intravitreal bevacizumab was well tolerated and associated with an improvement in VA, decreased retinal thickness, and a reduction in angiographic leakage in most patients, the majority of whom had been treated previously with PDT and/or pegaptanib [14].

In the years following these initial reports, the efficacy and tolerability of intravitreal bevacizumab was reported by hundreds of articles. It has been a commonly held belief by many clinicians that the efficacy of intravitreal bevacizumab and ranibizumab is comparable, with no clear difference, between the two treatments for most patients other than price; one intravitreal dose of ranibizumab is at least 40 times the cost of bevacizumab. The 1-year results of the Comparison of Age-Related Macular Degeneration Treatment Trials (CATTs) will be discussed in detail later in this monograph.

## 4. Ranibizumab

Ranibizumab is an antibody fragment that binds to and inhibits all identified VEGF isoforms (Figure 1). It was engineered to have 100 times the binding affinity of bevacizumab, despite having only a single-binding site. Given the absence of the Fc segment, the antibody fragment was designed to possess a shorter systemic half-life, improved retinal penetration, and less of a theoretical inflammatory reaction compared to bevacizumab [15].

Regarding a pharmokinetic comparison of bevacizumab and ranibizumab, when injected into human vitreous, 1-million-fold excess of either drug is present. The biologic activity in the vitreous of nonvitrectomized eyes has been demonstrated to last between 27 and 38 days after intravitreal injection of bevacizumab and 30 days after intravitreal injection of ranibizumab. The binding activity of bevacizumab is less than ranibizumab after a single injection. Both drugs penetrate the retina, but bevacizumab has a longer half-life in vitreous (as documented in rabbits and calculated in humans) and has been found in the serum and fellow eye in rabbits following intravitreal injection [16, 17]. All anti-VEGF agents administered intravitreally and in clinical use today have been identified in the systemic circulation [18, 19].

The phase III Minimally Classic/Occult Trial of the Anti-VEGF Antibody Ranibizumab in the Treatment of Neovascular Age-Related Macular Degeneration (MARINA) Trial evaluated the efficacy and safety of ranibizumab for the treatment of minimally classic or occult with no classic CNV associated with AMD. This 2-year, prospective randomized, double-masked, sham-controlled trial enrolled 716 patients from 96 sites in the United States. Patients were randomized in a 1 : 1 : 1 ratio to receive intravitreal ranibizumab at a dose of either 0.3 mg or 0.5 mg or sham injection monthly in one eye for 2 years.

At 24 months, 92% of patients who received 0.3 mg of ranibizumab and 90% of patients who received 0.5 mg ranibizumab lost fewer than 15 letters, compared with 52.9% in the sham group. The proportion of patients who gained at least 15 letters on the Early Treatment of Diabetic Retinopathy Study (ETDRS) chart from baseline to 24 months was 33.3% in the 0.5 mg group, 26.1% in the 0.3 mg group, and 3.8% in the sham group. The mean change in ETDRS VA from baseline to 24 months was a gain of 6.6 letters in the 0.5 mg group, a gain of 5.4 letters in the 0.3 mg group, and a loss of 14.9 letters in the sham-injection group [20].

The Anti-VEGF Antibody for the Treatment of Predominantly Classic Choroidal Neovascularization in Age-Related Macular Degeneration (ANCHOR) Trial was a multicenter, randomized double-blind trial that enrolled 423 patients to compare the efficacy and safety of ranibizumab and PDT with verteporfin in patients with predominantly classic CNV associated with neovascular AMD. Patients were assigned randomly to receive either 0.3 or 0.5 mg of ranibizumab plus sham verteporfin or sham intravitreal injection plus active verteporfin therapy. Ranibizumab or sham intravitreal injections were given monthly, and the verteporfin or sham was administered on day 0 and then as needed at months 3, 6, 9, and 12. 

At 12 months, 94.3% of patients in the 0.3 mg group and 96.4% in the 0.5 mg group lost fewer than 15 letters from baseline compared with 64.3% in the verteporfin group. The proportion of patients who gained at least 15 letters from baseline to 12 months was 40.3% in the 0.5 mg group, 35.7% in the 0.3 mg group, and 5.6% in the verteporfin group. The mean change in ETDRS visual acuity from baseline to 12 months was a gain of 8.5 letters in the 0.3 mg group, a gain of 11.3 letters in the 0.5 mg group, and a loss of 9.5 letters in the verteporfin group [21]. Rates of serious ocular or systemic adverse events were low in both trials [20, 21].

The results of these two trials documented dramatic vision improvement with mandatory monthly dosing regimens. Subsequently, the PIER and EXCITE phase IIIb studies sought to determine the dosing regimen that yielded the best VA outcomes while minimizing treatment burden. The results of these two trials supported the increased efficacy of monthly ranibizumab and the importance of timely treatment and demonstrated that regimented, quarterly dosing does not yield desirable VA outcomes [22, 23].

## 5. Individualized Anti-VEGF Therapy

### 5.1. Ranibizumab: As-Needed Regimen

The Prospective OCT Imaging of Patients with Neovascular AMD Treated with Intraocular Ranibizumab (PrONTO) study was an open-label, prospective, uncontrolled study that investigated a variable-dosing regimen for the treatment of wet AMD with intravitreal ranibizumab over two years. Thirty-seven patients received 3 consecutive monthly injections of 0.5 mg ranibizumab and were then followed monthly and retreated if there was an increase in OCT central retinal thickness (CRT) of at least 100 microns or a loss of best-corrected ETDRS VA of 5 letters or more. During the second year, the retreatment criteria were amended to include retreatment if any qualitative increase in the amount of fluid was detected on OCT. At 24 months, mean visual acuity improved by 11 letters with an average of 9.9 injections. In the PrONTO study, VA outcomes were comparable with those reported in ranibizumab phase III clinical studies, but with fewer intravitreal injections [24].

Results from the SAILOR (Safety Assessment of Intravitreal Lucentis for age-related macular degeneration) study, a Phase IIIb study of Lucentis (Ranibizumab, Genentech, South San Francisco, Calif.) for patients with all subtypes of new or recurrent active subfoveal wet AMD, suggest that quarterly visits were insufficient to monitor and capture disease progression [25, 26].

The SUSTAIN trial assessed the safety and efficacy of ranibizumab in patients with subfoveal CNV secondary to AMD using a dosing regimen individualized to patient characteristics. This open-label study recruited patients with subfoveal CNV secondary to AMD who were either ranibizumab-naïve or had completed treatment with ranibizumab or verteporfin PDT in the ANCHOR trial. Patients received three consecutive monthly injections of ranibizumab 0.3 mg (or 0.5 mg for the ANCHOR patients) (the “loading phase”), followed by monthly monitoring visits. Further treatment was administered if VA decreased by >5 letters or if central retinal thickness (CRT) increased by >100 *μ*m. A total of 513 patients were enrolled into the study and 69 patients who were ranibizumab-naïve have completed 12 months of followup. Compared with baseline, mean VA at month 12 increased by approximately 7 letters. Over 12 months, the mean standard deviation (SD) number of ranibizumab injections received by these ranibizumab-naïve patients was 5.3 (2.2), including the three “loading” injections. This study demonstrated that flexible, guided dosing with fewer ranibizumab injections and monthly monitoring can maintain efficacy outcomes in at least some patients [26].

The HORIZON study was an open-label multicenter extension study that included 853 patients (600 had been previously treated with ranibizumab initially, 184 had crossed over to treatment with ranibizumab, and 69 had not been treated with ranibizumab) who had completed one of the three 2-year, randomized, controlled trials of monthly intravitreal ranibizumab treatment (MARINA, ANCHOR, or FOCUS trial). Of 853 patients, two-year VA data were available for 384. These patients could receive 0.5 mg ranibizumab at 30-day or longer intervals as needed. Of the patients who received initial treatment with ranibizumab during the ANCHOR, MARINA, and FOCUS trials, there was a mean 10.2-letter increase in VA during the first 2 years of the studies. Patients that did not receive anti-VEGF therapy in the ANCHOR, MARINA, and FOCUS trials had worse outcomes. During the first year of the HORIZON study and the third year of the original trials, there was a 5.1-letter loss. The initial VA increase decreased by a mean of 8 letters with less frequent dosing in years 3 and 4. During the as needed dosing phase, the mean number of injections in the group initially treated with ranibizumab was 3.6. Patients in the treated crossover group received a mean of 4.2 injections. The results of the HORIZON trial demonstrate that a delay in the initiation of treatment is associated with poorer visual outcomes and continued but less frequent dosing in years 3 and 4 was associated with visual decline [27].

The as-needed ranibizumab studies have their strengths and weaknesses. For example, strengths of PRONTO and SUSTAIN include monthly follow-up, but the PRONTO trial has only a small cohort of patients. The SAILOR trial is the largest, but mandated only quarterly followup visits. Overall, these studies support frequent followup and individualized retreatment to achieve the best visual acuity gains with the as-needed treatment regimen as an alternative to the traditional monthly treatments used in the ANCHOR and MARINA trials.

### 5.2. Ranibizumab: Treat-and-Extend Regimen

The treat-and-extend regimen was first described by Freund et al. for the treatment of retinal angiomatous proliferation with an anti-VEGF agent. Treat-and-extend dosing regimen involves increasing intervals between treatment up to 10 weeks as long as no fluid is present on OCT. If fluid is present, the interval between treatments is shortened. Of note, Engelbert et al. first treated patients with 3 loading doses [28]. The treat-and-extend regimen can be quite variable in terms of treatment criteria, which can include vision loss and macular hemorrhage [28], and the length of time between treatment, which can extend up to 12 weeks [29, 30]. To date, no large, randomized, prospective trials have investigated the efficacy of the treat-and-extend regimen compared to the as-needed protocol, although this strategy is used currently in clinical practice.

Oubraham et al. compared two ranibizumab retreatment strategies, as-needed and treat-and-extend, in a retrospective review of 90 patients, 52 in the as-needed group, and 38 in the treat-and-extend group. Their treatment regimen included 3 loading doses for the as-needed group. Macular hemorrhage without fluid on OCT was not part of their retreatment criteria. They found that at one year, mean gain in VA was greater in the treat-and-extend group than in the as-needed group (+10.8 versus +2.3 letters, resp.). Eyes in the treat-and-extend group received significantly more mean injections (7.8 versus 5.2). Patients in the as-needed group were followed every 4-5 weeks. The number of follow-up visits was similar in both groups (8.5 versus 8.8) [29].

Gupta et al. compiled a retrospective, interventional, case series of 92 eyes managed with the treat-and-extend ranibizumab regimen. After 2 years, 32% had gained at least 3 lines of vision and received 8.36 and 7.45 injections during the first and second years, respectively. The treat-and-extend regimen was associated with fewer patient visits, injections, and direct annual medical costs compared with monthly injections [30].

### 5.3. Bevacizumab: As-Needed Regimen

The ABC trial is a prospective, double-masked, multicenter, randomized-controlled trial of 131 patients randomized to 3 loading doses of bevacizumab at 6-week intervals followed by as-needed treatment at six week intervals or an alternate treatment at the start of the trial (PDT, pegaptanib, or sham). Thirty-two percent of patients in the bevacizumab group gained at least 15 letters with a mean VA increase of 7 letters with a median of 7 injections. There was a mean decrease of 9.4 letters in the standard treatment group [31]. Several smaller, retrospective studies note a substantial improvement in VA with a protocol of three loading doses and then retreatment based mostly on OCT findings [32, 33].

Retrospective studies have demonstrated stabilization or improvement in VA following as-needed treatment with bevacizumab without a loading phase [34–36]. A prospective, open-label, nonrandomized clinical study reported a mean VA gain of 8.6 letters in 51 eyes after their second year of as-needed bevacizumab treatment with a mean of 1.5 injections given during year 2 [37].

### 5.4. Bevacizumab: Treat-and-Extend Regimen

Gupta et al. reviewed 166 eyes of 159 patients in a retrospective case series of patients treated with a treat-and-extend regimen of bevacizumab or ranibizumab for an average followup of 1.5 years. Patients had received monthly injections until dry on OCT. Treatment intervals were extended by 2 weeks at a time unless fluid was observed on OCT. Visual acuity outcomes and recurrence rates were similar for patients who received ranibizumab or bevacizumab. At 1 year, 32–35% of patients gained at least 3 lines of vision and 45–52% of patients had no recurrence over 1.5 years. Bevacizumab had a longer mean period of extension compared to ranibizumab. The treat-and-extend regimen was associated with lower medical costs when compared to the MARINA, ANCHOR, and PrONTO protocols, with a reduced mean number of injections and lower medical costs in the bevacizumab group [38].

## 6. Comparison of AMD Treatment Trials (CATTs)

To compare the efficacy of intravitreal injections of bevacizumab and ranibizumab and two dosing regimens, monthly and as-needed with monthly evaluation, the Comparison of AMD Treatment Trials (CATTs) were initiated. These multicenter, single-blind, noninferiority trials collectively enrolled 1208 patients with wet AMD. Retreatment was performed if at least one of the following criteria was met: fluid present on time domain OCT, decreased VA as compared to previous exam, new or persistent hemorrhage, or dye leakage or increased lesion size on fluorescein angiography. The primary outcome measure was mean change in VA at one year. After one year, there was no significant difference in VA outcomes between monthly bevacizumab and monthly ranibizumab and between as-needed bevacizumab and as-needed ranibizumab. Ranibizumab as-needed was found to be equivalent to monthly ranibizumab, but the comparison between bevacizumab as-needed and monthly bevacizumab was inconclusive [39]. This could be due to the less durable treatment effect of bevacizumab in a subgroup of patients [40].

The monthly ranibizumab regimen was associated with the greatest decrease in OCT central retinal thickness. Although not powered sufficiently to compare adverse event rates associated with the two drugs, the rates of death, arteriothrombotic events, and venous thrombotic events were similar for patients receiving bevacizumab or ranibizumab. The rate of serious systemic adverse events, primarily hospitalizations, was higher among the patients who had received bevacizumab, but rates of adverse events did not increase with increased exposure to the drug [39].

The results of the second year of the trial and the 5 similar trials currently being conducted in Europe may help to refine these data and could clarify lingering questions regarding adverse events.

## 7. Management of Nonresponders

As many as 10% of patients can lose a significant amount of vision despite 2 years of monthly anti-VEGF therapy [20, 21]. Within this group of individuals exist not only those who progress to disciform scar, RPE rip, massive subretinal hemorrhage, and geographic atrophy, but also eyes that demonstrate persistent macular fluid/blood and leakage on fluorescein angiography and vision loss. This small subgroup of patients is commonly referred to as anti-VEGF nonresponders. More aggressive forms of wet AMD, including retinal angiomatous proliferation (RAP), tachyphylaxis to anti-VEGF agents, mimics of wet AMD [41], and underlying genetic differences among patients may contribute to variability in anti-VEGF treatment response [42, 43].

The therapeutic approach can be tailored in these patients to include alternating bevacizumab and ranibizumab every 2 weeks to allow sustained anti-VEGF blockade, or combination therapy including PDT and/or intravitreal corticosteroids [41].

## 8. VEGF Trap-Eye

**Figure 2:**
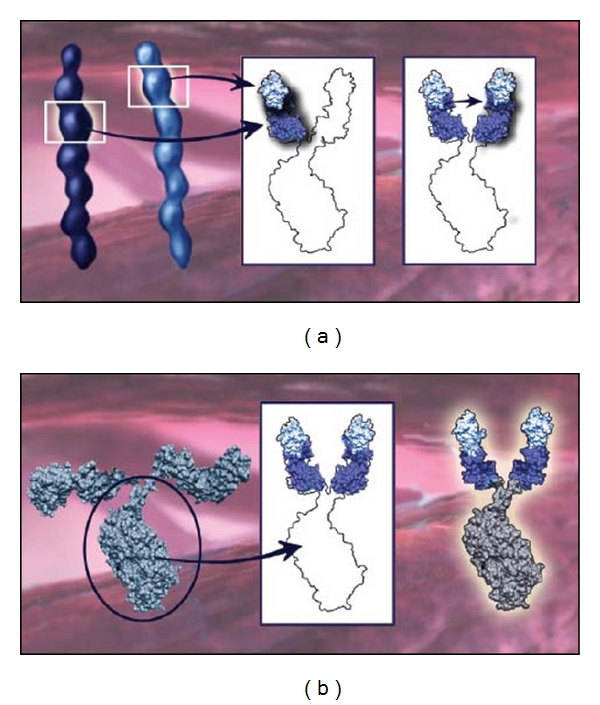
(a) A binding domain of VEGFR1 and VEGFR2 are fused to create 2 dual-domain arms for each VEGF Trap-Eye molecule. (b) The Fc portion of the IgG is fused to the two dual-domain arms to create the VEGF Trap-Eye molecule (see [9]).

VEGF Trap-Eye (EYLEA; Regeneron, Tarrytown, NY, USA) (VTE) is a soluble fusion protein consisting of 2 extracellular cytokine receptor domains and a human Fc region of immunoglobulin G (IgG). VEGF Trap-Eye includes specific extracellular components of VEGF receptors 1 and 2 fused to the constant region (Fc) of IgG1, resulting in 2 identical arms, each constructed from segments of both VEGFR1 and VEGFR2 (Figure 2). These components were selected based on their high affinity for both VEGF-A and placental growth factor (PlGF). The molecule uniquely binds both ends of activated dimerized VEGF or PlGF between its arms, preventing it from binding to the native receptors or cross-linking. The binding affinity of VEGF Trap to VEGF is 10 times higher than bevacizumab. The 2 mg dose of VTE at 83 days has been proven to have a similar biologic activity to ranibizumab at 30 days. The Fc portion likely plays a role in prolonging the half-life of the drug and could yield a longer duration of action.

VIEW1 was a phase III noninferiority trial conducted in North America that randomized 1217 patients to VTE 0.5 mg monthly dosing (0.5q4wk), VTE 2 mg monthly (2q4wk), VTE 2 mg every two months following 3 monthly doses (2q8wk), or ranibizumab 0.5 mg monthly (Rq4wk). The primary endpoint was the proportion of patients who lost fewer than 15 ETDRS letters from baseline to week 52. Secondary endpoints included mean change in BCVA at week 52. The following percentage of participants in the Rq4wk, 2q4wk, 0.5q4wk, and 2q8wk treatment arms gained at least 15 letters: 31%, 38%, 25%, and 31%, respectively. The proportions of patients maintaining vision at 52 weeks were 94.4%, 95.9%, 95.1%, and 95.1% for Rq4wk, 0.5q4wk, 2q4wk, and 2q8wk, respectively. All VTE groups were non-inferior to ranibizumab. Mean improvements from baseline to week 52 in ETDRS letter score was 8.1, 6.9, 10.9, and 7.9 letters for Rq4wk, 0.5q4wk, 2q4wk, and 2q8wk, respectively. 2q4wk was associated with a significantly greater mean VA improvement from baseline to week 52 than Rq4wk. Differences between other VTE groups and Rq4wk were nonsignificant. The difference in the mean reduction in central retinal thickness was not significant among the groups. The incidence of adverse events was similar across all treatments, with no increase in blood pressure noted. Overall, dosing monthly or every two months with VTE was non-inferior to monthly ranibizumab and was well tolerated [44]. The VIEW 2 study enrolled 1240 patients from Europe, Latin America, Asia, and Australia and yielded similar results [45].

## 9. Conclusions

Over the past two decades, the treatment of neovascular AMD has been revolutionized with the discovery of anti-VEGF agents that have enabled patients to regain vision thought permanently lost to this potentially blinding disease. With the goal of maximizing VA and minimizing the frequency of intravitreal injections and associated risks of treatment, evidence-based management of wet AMD has evolved into individualized anti-VEGF therapy with frequent followup and retreatment. As a safer, and more cost-effective alternative to the traditional monthly treatments used in the ANCHOR and MARINA trials, two individualized anti-VEGF treatment regimens have been described, but neither has been proven superior to date: as-needed (or “prn”) therapy and the treat-and-extend strategy. Despite a paucity of evidence comparing the as-needed versus the treat-and-extend treatment regimens, as the treat-and-extend regimen is explored further, it could prove to be the most efficacious, cost-saving, and preferred protocol. The current evidence-based treatment strategy for the management of wet AMD supports the use of either bevacizumab or ranibizumab either monthly or with a more individualized treatment strategy with close followup. As second generation anti-VEGF agents become available and the stress on our healthcare systems intensifies, increasingly efficacious and cost-conscious treatment strategies will be essential.
